# Bibliographical

**Published:** 1884-11

**Authors:** 


### Bibliographical.

### THE FORMATION OF POISONS BY MICRO-ORGANISMS, A BILOGICAL STUDY OF THE GERM THEORY OF DISEASE, BY G. V. BLACK, M.D., D.D.S.

This is a work on this branch of science of more than ordinary interest, as it contains a resume, in the main, of the work that has been done in this direction, not only in this country, but in others as well. After making reference to the occasion of preparing this work, Dr. B. says, in his preface: "The second part was written because I had something to say that I thought ought to be said at the present time. Men who have been most instrumental in the development of this subject, have given us little else than experimental facts. These will satisfy the minds of very few, as most of us appreciate any subject more if we understand why these things are, and it is this 'why' that I have sought to supply."

The first part consists of three Lectures, the first of which presents the work of quite a number of discoverers and experimenters upon the Germ Theory of disease. The second Lecture continues the same line of thought, referring to the modes of propagation of Micro-Organisms—Listers Antiseptic Surgery—Direct Examination for Micro Organisms—Miasmatic Contagion—Infection of Experiments, etc. The third Lecture discusses the Amœboid Theory of Contagion—The Theory and Works of Pasteur and Koch. The fourth Lecture discusses the production of poisons by micro-organisms, in which the functions of digestion and absorption are well presented, especially so far as they bear upon this subject. The digestion by Bacteria of the higher plants, and their nutrition, receives attention. The fifth Lecture discusses the East Plant—Salivary Digestion of the Higher Animals—Resorption of the Roots of the Milk Teeth, or temporary teeth—The Relation of Soluble Ferments to Living Tissues. The sixth Lecture discusses the Waste Products—Life as a Force—Law of Formation of Waste Products—Waste Products of Higher Animals, and of Higher Plants, and of Bacteria. The seventh Lecture discusses more fully, poisons developed by micro-organisms. In respect to this subject, the suggestion is made that further investigation is needed. This work presents, in a very condensed form, various phases of the micro-organisms as pertaining to disease, so far as the subject is developed up to the present time. Perhaps, nowhere can the student of this subject find so comprehensive and clear a presentation of the subject as in this work. In its preparation, Dr. Black has well studied every thing that has been presented upon the subject, and we have no hesitancy in here suggesting that a careful reading of this little work will be of far more service to the general student than any attempt he might be able to make in the way of gathering up what has been written upon the subject by various theorists and experimenters. We therefore recommend the work to every student in our profession, and, indeed, to every one who has a desire to be well posted upon the present status of this very important subject.

